# Innovative particle standards and long-lived imaging for 2D and 3D dSTORM

**DOI:** 10.1038/s41598-019-53528-0

**Published:** 2019-11-29

**Authors:** Angelina Provost, Corentin Rousset, Laura Bourdon, Sarra Mezhoud, Emma Reungoat, Camille Fourneaux, Timothée Bresson, Marine Pauly, Nicolas Béard, Laura Possi-Tchouanlong, Boyan Grigorov, Philippe Bouvet, Jean-Jacques Diaz, Christophe Chamot, Eve-Isabelle Pécheur, Catherine Ladavière, Marie-Thérèse Charreyre, Arnaud Favier, Christophe Place, Karine Monier

**Affiliations:** 1 Univ Lyon, Université Lyon 1, Lyon, France; 2 CNRS USR3010, ENS de Lyon, Laboratoire Joliot-Curie, F-69364 Lyon, France; 3 INSERM U1052 CNRS UMR5286, Cancer Research Center of Lyon (CRCL), Centre Léon Bérard, F-69008 Lyon, France; 4 CNRS UMR5223, INSA de Lyon, Laboratoire Ingénierie des Matériaux Polymères (IMP), F-69622 Villeurbanne, France; 5 CNRS UMR5672, ENS de Lyon, Laboratoire de Physique, F-69364 Lyon, France; 6 CNRS UMS3444, INSERM US8, ENS de Lyon, SFR Biosciences, UCBL, F-69007 Lyon, France

**Keywords:** Imaging, Centrosome, Single-molecule biophysics, Single-molecule fluorescence, Nanofabrication and nanopatterning

## Abstract

Direct stochastic optical reconstruction microscopy (dSTORM), developed in the last decade, has revolutionised optical microscopy by enabling scientists to visualise objects beyond the resolution provided by conventional microscopy (200 nm). We developed an innovative method based on blinking particle standards and conditions for long-lived imaging over several weeks. Stable localisation precisions within the 10 nm-range were achieved for single virions and *in cellulo* 2D imaging of centrosomes, as well as their reliable reconstruction in 3D dSTORM.

## Introduction

Cell biology has greatly benefited from the development of multiscale optical microscopy, which was until recently limited to a 200 nm-resolution due to the diffraction limit. Indeed, several super-resolution techniques^1–5^, including direct stochastic optical reconstruction microscopy (dSTORM)^6^, have emerged in the last decade providing a 10-fold gain in resolution compared to conventional microscopy. Although extensively studied, particularly in the context of 2D imaging, dSTORM has not been fully exploited owing to: (i) short time-delay after sample mounting to collect blinking events, and (ii) the lack of an adequate method of calibration, especially for 3D implementation.

dSTORM is based on the localisation of individual fluorophores that stochastically oscillate between an ON (bright) and OFF (dark) state. Thanks to this “blinking” phenomenon, it is possible to use high concentrations of fluorophores that would otherwise produce overlapping signals. Improved image resolution compared to conventional optical microscopy is achieved through the use of computer-assisted analytical approaches.

The improved resolution is reached by (i) localising fluorophore positions on each images through fitting with a Gaussian profile of the observed Point Spread Function (PSF), with a precision related to photon number and background level, (ii) displaying a reconstructed image from locations accumulated on thousands of images. The achievable xy-resolution on the reconstructed image is typically 20 to 30 nm (with the brightest blinking fluorophores), while the z-resolution is generally around 50–80 nm.

In order to get an optimal fluorophore blinking phenomenon, it is necessary to use a special buffer^1,6,7^ containing a thiol reducer and a low oxygen concentration. This latter condition is typically obtained through the use of oxygen scavenging enzymes, such as the enzyme pair glucose oxidase/catalase in the presence of glucose. Although efficient, this enzymatically deoxygenated buffer rapidly acidifies^8^, leading to a reduction in fluorophore blinking^6^. Consequently, this short-lived buffer must be replaced every 2–3 h^9^, which is both time- and buffer-consuming. Therefore, an alternative buffer is highly desirable to enable long-lived super-resolution imaging up to several days/weeks, with optimal quality of image reconstruction.

Furthermore, in the context of 3D imaging, deciphering the third axial dimension of a cellular structure is often based on the acquisition of multiple images at different focal planes (z-stacking). For dSTORM, this approach would be highly time-consuming. Dedicated methods have thus been developed to obtain z-localisation from 2D acquisitions^10^: PSF engineering (astigmatism, phase-mask), evanescent wave (supercritical angle fluorescence, SAF^11^), multiplane, and interferometry. However, the quality and reliability of the final 3D image reconstruction is difficult to assess owing to the lack of commercially available fluorescent blinking calibration tools. Ideally, the latter should be well-controlled micrometric objects, easy to label with blinking fluorophores at their periphery, thereby in contact with the buffer.

Here we present: (i) a simple strategy to maintain an efficient blinking phenomenon up to several weeks (by replacing the enzymatic deoxygenation of the buffer with a physical deoxygenation), and (ii) the design of blinking particle standards to evaluate both this novel buffer independently of biological variability and the quality of 3D image reconstruction.

## Results

### Design of fluorescent blinking particles

**Figure 1:**
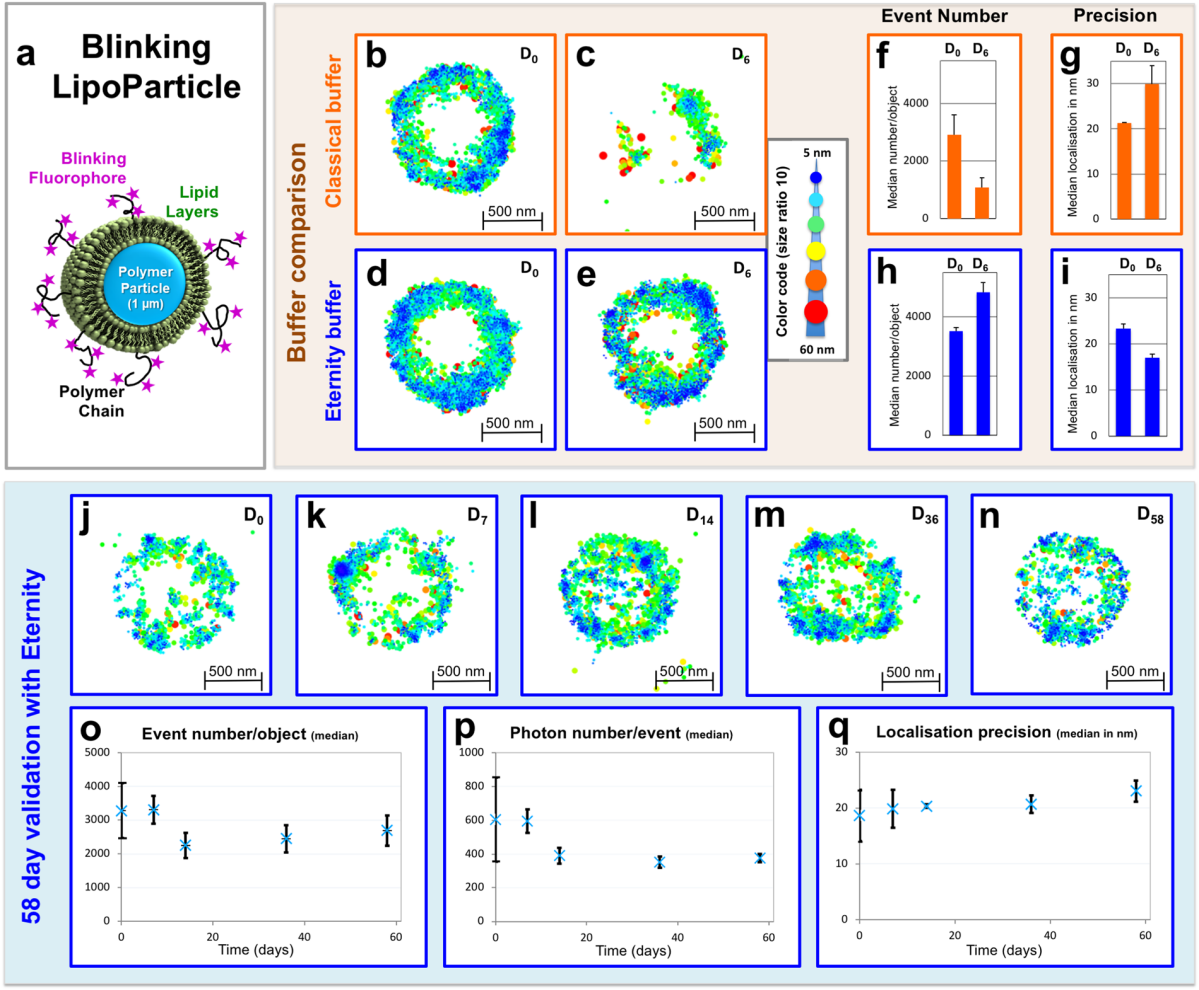
Long-lived- fluorescence dSTORM imaging of innovative blinking LipoParticles using the in-house developed Eternity buffer. (**a**) Schematic representation of our innovative blinking LipoParticle used for calibration purposes. The 1 µm polymer core in blue is surrounded by lipid layers (green) in which polymer chains (black line) bearing the fluorophores (purple stars) are immobilized. (**b**–**e**) Comparison of dSTORM LipoParticle reconstruction in classical (top) and Eternity (bottom) buffers at days 0 (**b**,**d**) and 6 (**c**,**e**). The dSTORM signals are colour-coded according to their localisation precision (5 to 60 nm, inverted rainbow scale). The size of the points is also proportional to their localisation precision within a ratio of 1/10 between the smallest and the biggest points. Measurements were conducted on LipoParticles (N = 3) from the same slide kept at 4 °C and in the dark throughout the entire experiment. (**f**–**i**) Number of blinking events (**f**,**h**) and median localisation precision (**g**,**i**), extrapolated from experiments b-e, and presented as bar charts at days 0 and 6. For each time point, N = 3 replicates (±SD). (**j**–**n**) Reconstruction by dSTORM of a LipoParticle at days 0 (**j**), 7 (**k**), 14 (**l**), 36 (**m**) and 58 (**n**). The same colour and size code as above was applied. Measurements were conducted on LipoParticles (N = 3) from the same slide kept at 4 °C and in the dark throughout the entire experiment. (**o**–**q**) The number of blinking events (**o**), the median number of photons *per* event (**p**) and the median localisation precision in nm (**q**) extrapolated from (**j**–**n**) are represented as a function of time. For each time point, N = 3 replicates (±SD). Table 1 summarizes the conditions used to acquire and visualise images in this figure.

With the view of elaborating fluorescent blinking standards, we developed LipoParticles composed of spherical polystyrene particles (1 µm diameter) surrounded by lipid layers^12,13^ (Fig. 1a), labelled with biocompatible fluorescent polymer chains exhibiting a lipid anchor^14^. The blinking fluorophore selected here was AlexaFluor647® (AF647), as it is known for its excellent blinking capacity^15^. The resulting lipid-polymer-AF647 probes (Supplementary Fig. 1) are water-soluble, smaller than the localisation precision (hydrodynamic radius in the 3 nm-range for a 20,000 g/mol polymer chain)^16^ and allow the fluorophore moiety to be at the LipoParticle surface in contact with the buffer. Successful LipoParticle labelling was assessed by images obtained on a widefield fluorescence microscope by merging two modes, namely epifluorescence and transmission (Supplementary Fig. 2).

### Long-lived 2D dSTORM imaging with the Eternity buffer

We initially used these surface-labelled LipoParticles to evaluate our physically-deoxygenated buffer (named Eternity buffer) in comparison with the classical enzyme-based buffer, over an experimental time-course of 6 days (Fig. 1b–i). Performance assessments of 2D dSTORM image reconstruction are dependent on the number of blinking events and on the localisation precision (directly related to the number of photons collected *per* blinking event). Implementation of the IGOR software facilitated the visualisation of 2D image reconstruction by coding the localisation precision of each event using colour and size (see Methods section). Blue and green signals exhibit the best localisation precision, while red and orange signals exhibit the worst localisation precision, thus probably reflecting in-focus (reddish) and out-of-focus (blueish) fluorophore position (Fig. 1b–e).

At D_0_, image reconstruction from 100,000 images was comparable for both buffers (Fig. 1b vs d), with a similar number of blinking events (Fig. 1f vs h) and a localisation precision around 20 nm (Fig. 1g vs i). However, at D_6_, only Eternity buffer provided an adequate image reconstruction (Fig. 1e), with a 20 nm (or even better) localisation precision (Fig. 1i). In the classical buffer, the number of blinking events decreased and the localisation precision worsened to around 30 nm (Fig. 1c,f,g).

Owing to this relevant result, the same LipoParticle sample in Eternity buffer was then used to monitor the quality of 2D dSTORM image reconstruction over two months (Fig. 1j–q). Interestingly, it was still possible to observe a significant blinking phenomenon at D_58_ (Fig. 1n), with a loss of both blinking event number and median number of photons not exceeding one third (Fig. 1o,p). We could also accurately reconstruct images with a localisation precision comparable to that at D_0_ (Fig. 1q).

Additionally, Eternity buffer is expected to be stable over a wide pH range, since comparable results are obtained at pH 5 and 8 (Supplementary Fig. 3) where most biological phenomena occur, whereas the enzyme-based buffer requires a pH of 8 for efficient blinking^8^. Eternity buffer is also compatible with the use of a focus-maintaining system on the microscope, since its refractive index is close to that of water, contrary to other buffers with higher indices^1^.

### Long-lived 2D dSTORM imaging of biological specimens using the Eternity buffer

To validate our buffer for biological applications, we next fluorescently-labelled several biological specimens with AF647-coupled probes prior to 2D dSTORM imaging in Eternity buffer.

**Figure 2:**
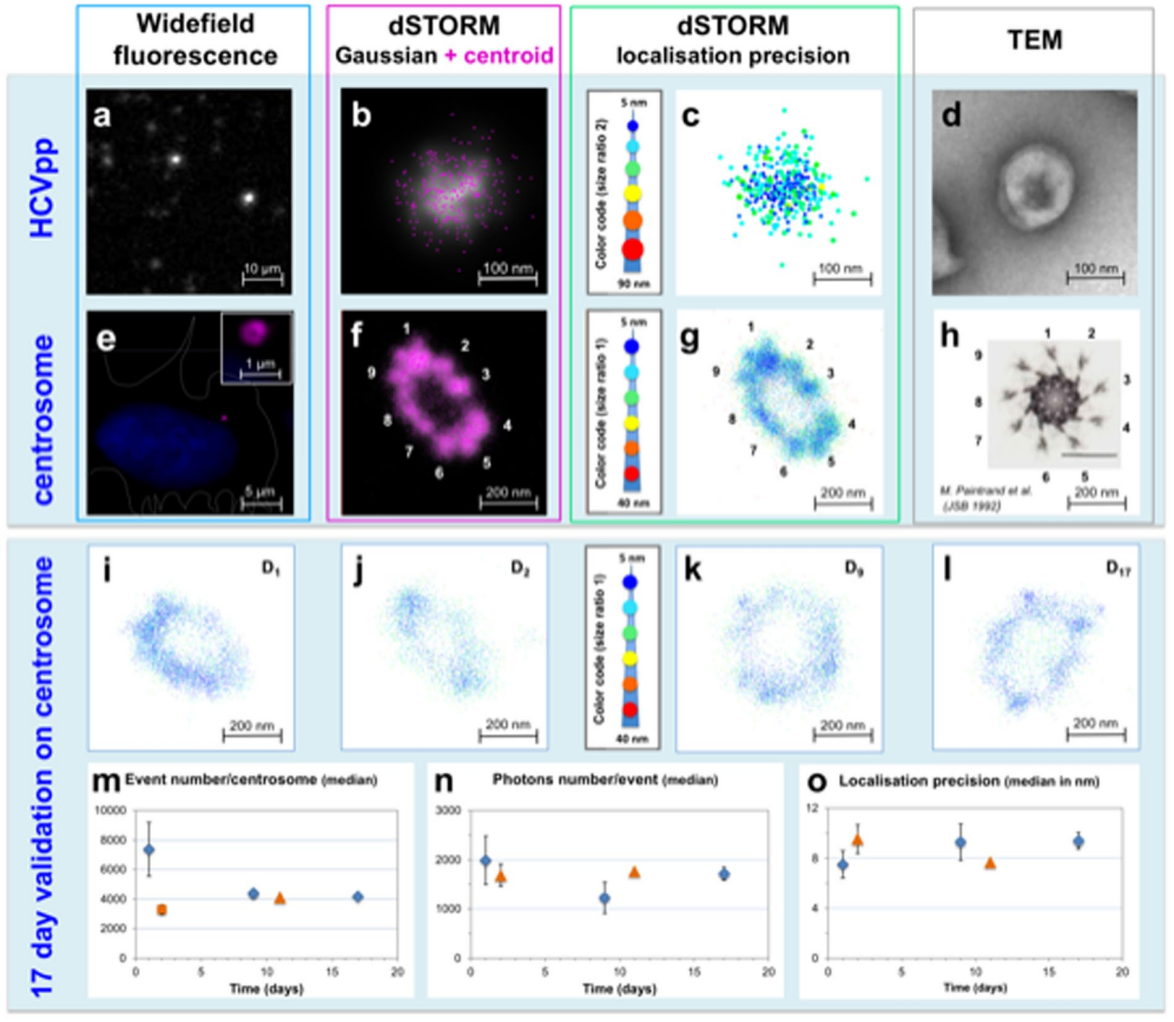
Evaluation of the Eternity buffer for 2D dSTORM imaging of various biological specimens labelled with AF647. (**a**–**c**) Widefield fluorescence (**a**) and dSTORM images of hepatitis C virus pseudoparticles (HCVpp) labelled with the lipid-polymer-AF647 probe. dSTORM images represent the merging of 15,000 images combining the centroid (purple) and gaussian width (white) of each point detected (**b**), and the colour (5 to 90 nm; inverted rainbow colour scale) and size (from a ratio of 1 to 2) coding of each point (**c**). (**d**) TEM image of a lipid-polymer-AF647-labelled HCVpp particle, negatively stained with phosphotungstic acid. (**e**–**g**) Fluorescence microscopy image of U2OS cells with DAPI-labelled nucleus (**e**, blue) and Cep164 protein immuno-detected with AF647-labelled secondary antibodies, to image mature centrosomes (purple) at the periphery of the nucleus. The shape of the cell is delineated by a white dotted line using the corresponding bright field image. Inset shows an enlarged view of the centrosome region illustrating the epifluorescence resolution obtained for this donut-like structure. dSTORM image of the mature centrosome (**f**) in which each point is represented by its centroid (purple points) and its gaussian width (white). IGOR representation of 2D dSTORM images (**g**) in which each point is colour-coded (5 to 40 nm; inverted rainbow colour scale) as a function of its localisation precision. (**h**) TEM image of another centrosome, previously imaged by Paintrand *et al*.^23^ at the plane of subdistal appendages, illustrating the well-known nine-fold symmetry of this structure. (**i**–**l**) Reconstruction by dSTORM of a centrosome with Cep164/AF647 staining at days 1 (**i**), 2 (**j**), 9 (**k**) and 17 (**l**), in which each point is colour-coded (5 to 40 nm; inverted rainbow colour scale) as a function of its localisation precision (**m**–**o**). The number of blinking events *per* centrosome (**m**), average number of photons *per* event (**n**) and median of localisation precision (**o**) are presented as a function of time for 50,000 images of Cep164 detected with AF647 on the same slide (blue diamonds, technical replicates) and on different slides (orange triangles, independent biological replicates). For each time point, standard deviations depict variations between 2 technical replicates on the same reference slide (day 1), 2 technical replicates on a biological replicate (day 2), 3 technical replicates on the same reference slide (day 9), 1 series on a second biological replicate (day 11), 3 technical replicates on the same reference slide (day 17). These conditions are depicted in Supplementary Figure 10. Table 2 summarizes the conditions used to acquire and visualise images in this figure.

The first biological model used was non-infectious enveloped viral particles (*i*.*e*. pseudoparticles) of the hepatitis C virus (HCVpp) (Fig. 2a–d). Their small size, 100 nm-diameter, is below the limit of resolution of conventional microscopes, arguing in favour of their detection by 2D dSTORM. Furthermore, we have recently shown that the HCVpp lipid envelope could be efficiently labelled with a lipid-polymer probe^17^; these viral particles therefore constituted a highly relevant model to test our buffer. HCVpp labelling with lipid-polymer-AF647 probe appeared stable, and provided a strong fluorescent signal with a very high contrast. TEM imaging confirmed that labelled and unlabelled viral particles had similar morphologies, displaying neither lysis nor aggregation (Supplementary Fig. 4). Therefore, the image shown in Fig. 2b is the first 20 nm-scale observation of HCV under environmental conditions, and enabled us to visualise individual virions, while only clusters are observable by conventional optical microscopy (Fig. 2a). This dSTORM technique combined with our long-lived imaging Eternity buffer will in the future provide visual data over several days. Our observations thus pave the way for dSTORM imaging of HCV virions and other enveloped viruses, in particular inside their host cells.

Having demonstrated both the efficacy of our Eternity buffer for long-lived 2D dSTORM imaging and validated the technique using viral particles, we were keen to test our methodology on centrosomes (Fig. 2e–h), a structure previously studied by other groups using different super-resolution approaches^18–22^. The centrosome is a key organelle part of the microtubule organizing centre (MTOC), with a nine-fold symmetry as revealed by TEM^23^. This symmetry is highlighted by 9 sub-distal appendages (Fig. 2h), where the Cep164 protein is localized. Here, Cep164 was initially detected in U2OS cells following primary and AF647-coupled secondary antibodies to label the mature centrosome^24^. By widefield microscopy, this labelling appears at the best as a single donut structure (inset Fig. 2e), while 2D dSTORM imaging enabled the visualisation (though not distinctly) of centrosome appendages in the Eternity buffer (Fig. 2f–g). These structures can be compared to the reference TEM image^23^ of an *in vitro* isolated centrosome, displayed at the same scale (Fig. 2h).

Optimisation of the blinking conditions was achieved by changing the thiol reducer^25^ in Eternity buffer (Supplementary Fig. 5). Switching from mercaptoethylamine (MEA) to β-mercaptoethanol (BME) reducer provided a significantly higher photon collection, resulting in a better localisation precision (Supplementary Fig. 5d,e). However, blinking is highly reduced after 22 days when Eternity buffer with BME is used. Therefore, further studies were carried on with Eternity buffer containing MEA.

By combining the finely tuned IGOR-based analytical approach and our Eternity buffer, centrosome appendages were visualised for up to 17 days on 3 independent biological samples, by 2D dSTORM reconstruction of 50,000 images (Fig. 2i–l). Data compilation of 10 centrosome structures revealed, starting at D_2_, a stable number of blinking events (Fig. 2m) with a constant number of photons over time (Fig. 2n), and a steady localisation precision below 10 nm (Fig. 2o). This demonstrates the long-lasting efficiency of the Eternity buffer for observing biological samples by dSTORM.

### Applying 2D dSTORM imaging to multicoloured biological specimen detection

A critical application of fluorescence imaging in biology is the possibility of staining various specimens/objects with different fluorophores to study their interaction/localisation.

We therefore challenged the robustness of our Eternity buffer by evaluating other red and far-red fluorophores (Supplementary Fig. 6). Although its performance was equivalent to that of a classical buffer for imaging AF647 at D_0_, we clearly observed that the Eternity buffer provided a higher photon collection (Supplementary Fig. 6a), as well as a better precision in xy and z (Supplementary Fig. 6b,c) for DL550 and DL650. We then applied two different primary/secondary antibody stains to RPE1 cells, in order to highlight the distal part of the mature centriole with Cep164 (combined to DL550) and the proximal part with the pericentrin (combined to AF647, Supplementary Fig. 7). To comply with our previous observations of long-lived imaging with the Eternity buffer, Supplementary Fig. 7 illustrates the double colour dSTORM reconstruction of the centrosome obtained at D_40_. This demonstrated that the Eternity buffer could be applied to long-lived multicoloured dSTORM imaging and is highly promising for biological applications requiring different fluorophores.

### 3D dSTORM imaging using the Eternity buffer and fluorescent standard LipoParticles

Finally, labelled LipoParticles were used to validate 3D dSTORM reconstructions of biological structures. The generation of 3D reconstructions from 2D dSTORM data sets was achieved using phase ramp imaging localisation microscopy (PRILM^26^). PRILM requires the use of a reference PSF, determined experimentally with TetraSpeck beads (see Methods for details and Supplementary Fig. 8a–c). However, this procedure may generate artefacts, leading to structural inconsistencies in the 3D reconstruction. To illustrate this, we generated a “non-optimised” PSF, with TetraSpeck beads loosely attached to the surface (Supplementary Fig. 8b).

**Figure 3:**
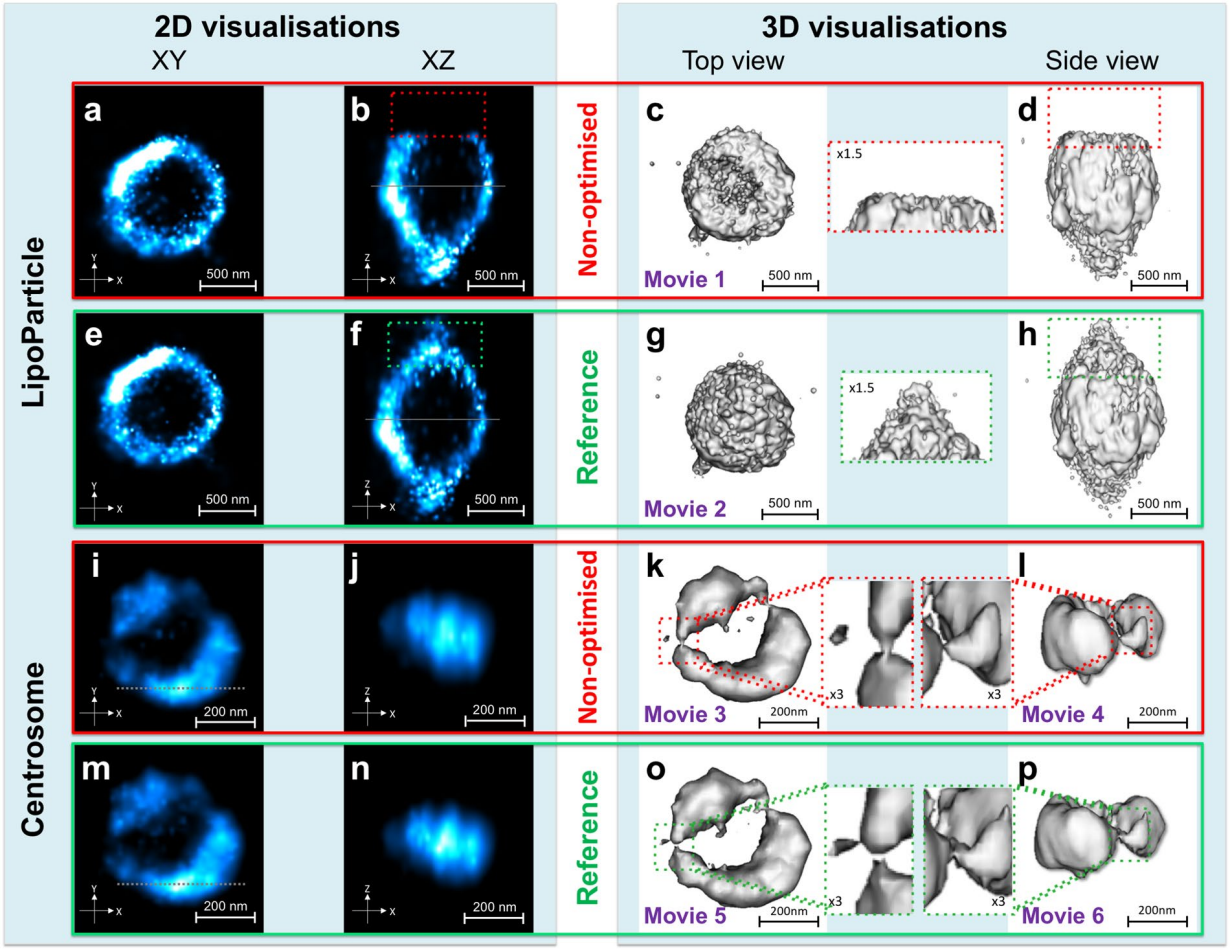
3D dSTORM image reconstruction using the LipoParticles as a calibration tool. (**a**–**h**) 3D dSTORM reconstructions using surface rendering mode (**c**,**d**,**g**,**h**) of LipoParticles visualised in 2D dSTORM (**a**,**b**,**e**,**f**) from density of points using the gaussian width (intensity varies from blue to white as a function of the density of points). 2D/3D dSTORM reconstructions with the non-optimised PSF (**a**–**d**), compared to the reference PSF (**e**–**h**). The xy views in (**a**) and (**e**) were generated at the height indicated by the solid line drawn respectively in xz views in (**b**) and (**f**). (**i**–**p**) 3D dSTORM reconstructions using surface rendering mode (**k**,**l**,**o**,**p**) of centrosomes visualised in 2D dSTORM (**i**,**j**,**m**,**n**) from the density of points using the gaussian width (intensity varies from blue to white as a function of the density of points). 2D/3D dSTORM reconstructions with the non-optimised PSF (**i**–**l**), compared to the reference PSF (**m**–**p**). The xz views in (**j**) and **(n**) were generated at the height indicated by the dotted line drawn respectively in (**i**) and (**m**). The dotted squares indicate the same area and are enlarged for a better observation. LipoParticles are labelled with the lipid-polymer-AF647 probe, and centrosomes in U2OS cells with primary antibodies directed against Cep164, followed by secondary antibodies labelled with AF647. The dataset is representative of the results obtained in 3 independent datasets obtained of LipoParticles. Table 3 summarizes the conditions used to acquire and visualise images in this figure.

The LipoParticles 3D image reconstructions obtained from both PSFs were then compared (Fig. 3a–h, Supplementary Fig. 8d–f). For LipoParticles, the “non-optimised” PSF systematically led to truncated reconstructions (Fig. 3a–d and Movie 1), whereas the reference PSF enabled complete 3D reconstructions (Fig. 3e–h and Movie 2).

An identical approach was then used to reconstruct the centrosome (Fig. 3i–p). Several structural details appeared different in 3D views (dotted boxes on Fig. 3k,l,o,p), yet choosing between the structures generated by each PSF seemed arbitrary (Fig. 3i–p, Movies 3–6). Since 3D reconstructions of LipoParticles, used as validation standards, appeared complete only when using the reference PSF, we speculated that this PSF could also be optimal for the centrosome. This enabled to discriminate between both centrosome 3D structures. The two grooves observed in the centrosome reconstructions (Fig. 3k,l,o,p) correspond to areas less dense in Cep164 labelling (Fig. 3o and inset). This could correspond to an area with a higher angle between subdistal appendages as described by Shi *et al*.^19^ and/or to the presence of a component with an asymmetric position. In conclusion, the combination of our buffer and fluorescent blinking LipoParticles in 2D dSTORM experiments paved the way for reliable 3D image reconstructions with a good level of reliability.

## Discussion

Overall, our study unveiled a physically-deoxygenated Eternity buffer enabling long-lived dSTORM imaging, validated by our blinking particle standards. This buffer provides scientists with a longer period of time for image acquisition and, therefore, more reliable computer-assisted image/statistical analyses than classical enzyme-based buffers that need to be replaced every 2–3 h. Indeed, we were able to carry out dSTORM acquisition on the same sample in Eternity buffer for up to 58 days, without any loss of image quality (stable localisation precision of 20 nm). This remarkable property of our buffer is an invaluable asset especially for long-term acquisition experiments, such as high-throughput studies with multiple conditions^9^ or multiple acquisitions of the same condition^27^.

Our Eternity buffer exhibits a greater capacity to remain at a constant pH (8.0) throughout the experiments, and permits an efficient blinking phenomenon at a low pH of 5, as opposed to the classical enzyme-based buffers^8^. We believe that this capacity is due to the innovative physical deoxygenation of our buffer, which does not rely on enzyme availability and does not result in an acidification of the environment. To sum up, our long-lived results obtained on various calibration and biological samples mounted with the Eternity Buffer and sealed with twinsil, ascertain that our experimental set-up is well suited to maintain a reduced oxygen level, in contrast to other reports exhibiting a fast re-oxygenation using an enzymatic buffer^28^. This is attributed to both our mounting set-up that limits the contact surface with air (Supplementary Fig. 8d) and to the fact that enzymatic buffers eliminate oxygen, rather than replacing it, thereby inducing a reduced pressure inside the sealed cavity that favors air leak and thus fast re-oxygenation. Moreover, Eternity buffer is also compatible with evanescent/TIRF microscopy and the use of a focus-maintaining system, since its refractive index is close to that of water, contrary to other buffers with higher indices^1^; this property enables an improved image quality.

Image rendering of super-resolution images remains a challenge in the single molecule localisation microscopy field. The goal of the module we developed using the IGOR software, a software enabling scientific graphing and calculation, was to implement another dimension for 2D dSTORM data, reflecting the localization precision of each detected event. The size (and/or colour) of each point is then proportional to the value of the localization precision for each x,y and z dimension (see method section). Optimising the number of acquired images for each data set is also a matter of intense research since a compromise must be found between a sufficient fluorophore density and an acceptable acquisition time/storage space (Supplementary Fig. 9).

We then demonstrated the versatility of this buffer for dSTORM imaging of distinct biological specimens, namely hepatitis C virus pseudoparticles (HCVpp) of about 100 nm-diameter, and mature centrosomes *in cellulo*. For the former model, the image shown in Fig. 2b is the first 20 nm-scale observation of HCV under environmental conditions *(i*.*e*. normal atmospheric pressure and aqueous buffer).

The relevance of our Eternity buffer was further confirmed by successively performing long-lived 2D dSTORM imaging of mature centrosomes within U2OS cells for up to 17 days on the same sample, and RPE1-derived centrosomes for up to 40 days using two different antibody/fluorophore combinations. Visualising both the distal and the proximal part of the centrosome with known markers (Supplementary Fig. 7) will constitute a valuable asset to collect higher number of structures during the long-lived dSTORM imaging in the Eternity buffer, and to analyse the biological variability of the centrosome architecture.

Our second innovation is the design of blinking particle standards to validate the quality of both 2D and 3D dSTORM image reconstructions. We elaborated original micrometric labelled LipoParticles, the fluorophores located at their periphery being in contact with the buffer. As such, the labelled LipoParticles were crucial for discriminating between truncated and optimal 3D image reconstructions of centrosomes. These well-controlled particle standards are especially relevant for 3D dSTORM since they exhibit a spherical shape and are detectable independently of transient binding of fluorescent molecules^29^. These spherical blinking LipoParticles constitute key nano-objects for 2D and 3D dSTORM validation, playing the role of rulers, essential for metrology aspect in the validation of dSTORM workflows.

Finally, the simplicity of use of the innovative strategies presented herein, independently and in combination, alongside the improved localisation precision obtained for 2D and 3D dSTORM imaging, render these strategies highly promising and pose them as essential tools for future microscopy localisation studies in biology.

## Methods

### Materials

All chemicals were purchased from Sigma-Aldrich, Acros and Fluka at the highest level of purity available, and used without further purification. Solvents (Fisher Scientific), the phospholipids 1,2-dipalmitoyl-*sn*-glycero-3-phosphocholine (DPPC) and 1,2-dipalmitoyl-*sn*-glycero-3-phosphate sodium salt (DPPA) (Avanti Polar Lipids), and the fluorophore AlexaFluor 647 (AF647) cadaverine di-sodium salt (Life Technologies SAS) were used as supplied. Lipid-P(NAM-*co*-NAS) copolymer chains were synthesized as previously described^30^ from a lipid RAFT agent^31^ (*Mn* = 20,300 g/mol and Đ = 1.04). All solvents used for determining photophysical properties were of spectrophotometric grade.

### Synthesis of the fluorescent polymer probe (lipid-polymer-AF647)

11.6 mg of lipid-P(NAM-*co*-NAS) copolymer (0.57 × 10^−3^ mmoles of chains corresponding to 30.2 × 10^−3^ mmol of NAS units) were dissolved in 0.25 mL of dimethylformamide (DMF). Then, 3 mg (3 × 10^−3^ mmol) of AF647 fluorophore in DMF were added with 1 molar equivalent of triethylamine (TEA). Polymer concentration was adjusted to 7 mg/mL with DMF and the binding reaction was carried out at 40 °C in the dark under stirring. The kinetics of fluorophore binding was followed by size exclusion chromatography (SEC) (see description below) and reached a plateau with a maximum yield of 60% after 4 days, corresponding to an average number of 3.05 fluorophores per polymer chain. The residual activated ester units along the chain were hydrolysed at room temperature by adding 10 mL of borate buffer (50 mM, pH 9.0) for 2 days, leading to a negatively charged polymer backbone (carboxylate groups).

The fluorescent polymer probe was purified by dialysis (Spectrum Labs, Spectra/Por^®^7, MWCO: 10,000 g/mol) against a 0.5 M NaCl solution (2 baths), enabling the rapid release of the free AF647 fluorophore, then against deionized water (4 baths) and milliQ water (two baths). The cyan-blue-coloured lipid-polymer-AF647 probe was dried by lyophilisation.

### Characterisation of the polymer probe

#### SEC with online UV-visible and refractive index (RI) detectors

SEC was used to monitor the binding yield of the fluorophore onto the copolymer according to a previously described method^32^, with a Waters 1515 isocratic HPLC pump (flow rate: 1 mL /min) and a Styragel HR4E Waters column at 30 °C. The eluent was DMF with LiBr (0.05 mol/l). Detection was provided by a Waters 2410 RI and a Waters 2489 UV-visible detector set at 650 nm (AF647 maximum absorption wavelength in water is 649 nm). Analyses were performed by injecting 10 µL of the reaction medium (diluted to 5 mg/mL with DMF). Data acquisition and treatment were carried out using the Breeze software (Waters).

#### UV-visible absorption and fluorescence emission spectra

UV-visible absorption spectrum of the lipid-polymer-AF647 probe was recorded on a Shimadzu UV-2600 spectrophotometer at room temperature using 1 cm quartz cell (integration time 0.1 sec). The fluorescence emission spectrum was recorded on a Horiba-Jobin Yvon Fluorolog-3® spectrofluorimeter at 298 K, using 1 cm quartz cell. The steady-state luminescence of diluted solutions was excited by unpolarised light from a 450 W xenon CW lamp and detected at a right angle (90°) by a red-sensitive Hamamatsu R928 photomultiplier tube (slits: excitation: 3 nm, emission: 1 nm; integration time: 0.1 sec). Spectrum was reference-corrected for both the excitation light intensity variation (lamp and grating) and the emission spectral response (detector and grating). For determination of the fluorescence quantum yield, reference was AF647 fluorophore. Excitation of reference and sample was performed at the same wavelength (590 nm).

Absorption and fluorescence emission spectra of the lipid-polymer-AF647 probe in water were very similar to those of the free fluorophore (Supplementary Fig. 1), with an emission band in the far-red region (650–750 nm). The molar extinction coefficient (ε) and the fluorescence quantum yield (Φ) were determined from the absorption and emission spectra, respectively. For the polymer probe, ε was more than twice that of the free fluorophore, and Φ was 55% of that of the free fluorophore. Brightness was determined from the product of ε and Φ.

### Synthesis of fluorescent blinking LipoParticles

LipoParticles were prepared by adding a preformed liposome dispersion to polystyrene particles (1 µm diameter, ammonium surface groups, *Polysciences*) following a previously described procedure^33^. The mixture was vortexed for 1 h at 70 °C and 1,300 rpm. Thereafter, in order to separate the LipoParticles from non-adsorbed lipids, the suspension was centrifuged at 9,000 × g for 10 min at 21 °C. The pellet containing LipoParticles was dispersed in pure water.

Preformed liposomes were prepared via a “hydration of a thin *lipid* film” process, the so-called *Bangham method*. Homogenisation of size was performed by sonication^33^ or extrusion treatments^34^. The lipid molar formulation used was either 89.9/10/0.1 (Supplementary Fig. 2), 89/10/1 (Supplementary Fig. 9), or 89.97/10/0.03 (all other figures) of DPPC/DPPA/lipid-polymer-AF647. Lipid-polymer-AF647 probe was solubilized in a 65/35 chloroform/ethanol v/v or 71/28/1 chloroform/ethanol/water v/v/v mixture.

### Observation of the fluorescent LipoParticles by brightfield and fluorescence microscopy

Observation of the LipoParticles was performed on a widefield fluorescence microscope Leica DMI6000B using an excitation source Leica Halogen Bulb and an EMCCD camera (Hamamatsu C9100, 512 × 512 pixels, pixel size: 16 µm). A 100x oil immersion objective lens (HCX PL APO Leica, NA = 1.46) was used for both brightfield and fluorescence. A BGR filter (450/90, 502/15, 590/20) was used for fluorescence. On the glass coverslip (thickness 170 ± 5 µm), 10 µL of sample and 2 µL of a 5 M NaCl solution were mixed, to decrease the Debye length and favour adhesion of the charged LipoParticles onto the coverslip. The images were acquired with the LASAF software (Leica) to manage the microscope and the Wasabi software (Hamamatsu) to pilot the EMCCD camera. Images were processed using the ImageJ freeware.

### Production of HCV pseudoparticles (HCVpp) and labelling with the fluorescent polymer probe

In order to bypass limitations due to the use of infectious viruses, we used a surrogate model of HCV virions, *i*.*e*. pseudotyped particles (HCVpp). They are easy to produce in cell cultures, at high rates and with optimal reproducibility between batches^35^. HCVpp are assembled onto a retroviral core, harbour the viral glycoproteins E1-E2 at their surface (embedded in the lipid envelope), and are most suitable to track viral entry events^36,37^.

HCVpp of genotype 1a (H77; AF011752) were produced as previously described^35–38^, from 293 T cells co-transfected using the calcium phosphate method with a murine leukaemia virus (MLV) Gag-Pol packaging construct, an MLV-based transfer vector encoding GFP as a reporter protein, and the E1–E2 expression constructs. Supernatants containing pseudoparticles were collected 48 h post-transfection and filtered on 0.45 μm. Pseudoparticles were concentrated 100-fold by ultracentrifugation through a 20% sucrose cushion at 75,000 × g for 2 h at 4 °C. Pellets were then resuspended in culture medium without foetal calf serum.

Concentrated HCVpp (200 μl, infectious titres ranging from 7 × 10^5^ to 2 × 10^6^ transducing units /mL, from one preparation to the other^35^) were incubated with 1 μM polymer probe (final) at 4 °C for 30 min, as described^17,39^. Viral particles were then placed onto a 20% sucrose cushion, topped with 1 mL phosphate-buffered saline (PBS) and ultracentrifuged at 135,000 × g for 1 h 30 min at 4 °C in a TLA-100.4 Beckman rotor. Pelleted labelled virions were then collected immediately after centrifugation, resuspended in PBS and stored at 4 °C in the dark. Handling of the lipid-polymer probe for HCVpp labelling was greatly facilitated due to its water solubility.

### Observation of the labelled HCVpp by widefield fluorescence microscopy and TEM

Purified and labelled HCVpp were observed by: 1) fluorescence microscopy, and 2) TEM, to validate their integrity and morphology at the nanometric scale following their labelling with the fluorescent polymer probe.

#### Widefield microscopy

Widefield microscopy was carried out as described for the LipoParticles (see section “Observation of the fluorescent LipoParticles by brightfield and fluorescence microscopy”). Ten microliters of sample were placed onto a glass coverslip (thickness 170 µm).

#### Transmission electron microscopy

Observation by TEM after negative staining with phosphotungstic acid (2% w/v, adjusted to pH 7.0 just before use) was performed on a JEOL JEM 1400 microscope operated at 80 kV and equipped with a high port digital camera Gatan Orius 600 (CIQLE facility, SFR Lyon Est). Carbon-coated copper grids were purchased from Ted Pella Inc. Prior to their use, grids were submitted to glow discharging, followed by immediate sample deposition. Labelled HCVpp retained their integrity, and displayed a similar morphology as that of unlabelled HCVpp, with neither particle aggregation nor lysis (Supplementary Fig. 4).

### *In cellulo* centrosome labelling

U2OS cells (ATCC, HTB-96) were grown and maintained as described^24^ with Dubelcco’s modified Eagle medium without phenol red (ThermoFisher #31053-028). RPE1 cells (ATCC, CRL 4000) were grown at 37 °C in a 5% CO_2_ incubator in Dulbecco’s modified Eagle medium/F12 without phenol red (ThermoFisher #21041025), supplemented with 10% (v/v) fetal calf serum (FCS, Life Technologies, Inc.) and 1% penicillin/streptomycin solution (Life Technologies, Inc). One hundred thousand cells were plated as described^24^ onto clean and sterile glass coverslips (diameter 18 mm, thickness 170 ± 5 µm, quality 1.5 H, Marienfeld Superior #0117580), except for Figs 2i–o and 3, Supplementary Figs 5 and 6 in which cells were plated onto Willco GWST 35–22. Fixation and immunofluorescence steps were performed as described^24^ with the subsequent modifications. The following primary antibodies were incubated for 1 h at 37 °C: Cep164 and pericentrin as detailed^24^. The following secondary antibodies were then incubated for 2 h at 37 °C: F(ab)’2 GAR-AF647 at 1:100 (Invitrogen #A21246), and DAM-DL550 at 1:250 (Agrisera #AS12 193) or DAM-DL 650 at 1:100 (Agrisera #AS12 2304) and GAR-DL550 at 1:100 (Agrisera #AS12 1968). *In cellulo* centrosome labelling was imaged by widefield microscopy as described^24^.

### Preparation of dSTORM buffers

#### Classical dSTORM buffer

The classical buffer was prepared to reach a final concentration of 100 mM MEA hydrochloride (stock of MEA/HCl, 1 M, Sigma-Aldrich) in a solution of 50 mM Tris, 10 mM NaCl, 5% glucose (stock at 25% w/v, Sigma-Aldrich), 0.56 mg/mL Glucose oxidase (stock at 22 mg/mL, Sigma-Aldrich), 0.04 mg/mL catalase (stock at 5 mg/mL, Sigma-Aldrich) and was adjusted to pH 8.0. Buffers were spun down for 3 min at 16,000 X g and the supernatant alone was used to mount the coverslips.

#### The eternity buffer

It was prepared with 100 mM MEA hydrochloride (from 1 M stock of MEA/HCl in deionized water, Sigma-Aldrich) or 100 mM BME (from a 14.3 M stock, Sigma-Aldrich) in the same buffer solution as that described for classical dSTORM (50 mM Tris, 10 mM NaCl) and was adjusted to pH 8.0 (or pH 5.0 for experiments displayed in the Supplementary Fig. 3). A concentration of 10 mM of MEA hydrochloride was instead used for Fig. 1f,g, also seen in Supplementary Fig. 9b. Oxygen was removed by smooth bubbling of N_2_ for 20 minutes per volume of 1 mL. The buffer was immediately used to mount the coverslips.

### Mounting LipoParticles for dSTORM imaging

Ten microlitres were deposited onto a 35 mm Willco dish (GWST-3512). After adding 250 µL of classical dSTORM or Eternity buffer, a 18 mm round glass coverslip was placed and sealed with twinsil speed 22 (Picodent, ref 13001002). Microscopic observation and image acquisition were started within a couple of hours (Day 0). Coverslips were then stored in the dark at 4 °C until further observation.

### Mounting HCVpp AF647 slides for dSTORM imaging

#### Polylysine coating

Round coverslips (diameter 18 mm, thickness 170 ± 5 µm, quality 1.5 H, Marienfeld Superior, #0117580) were coated with 1% polylysine for 15 min and rinsed, before drying at 37 °C.

#### TetraSpeck beads incubation

Ten microliters of TetraSpeck beads (100 nm, ThermoFisher Scientific) diluted 200 times in distilled water, were vortexed, and deposited on a thin sheet of parafilm. The polylysine-coated face of the coverslip was then gently squeezed against the parafilm for 5 min at room temperature, and rinsed twice. This step proved to be crucial for automated drift correction during image analysis on the Zeiss Software, determined from the mean of 30 consecutive images (Image self alignment) and based on an auto-correlation method (“model-based”), irrespective of the sample observed.

#### Sample deposition

Viral particle suspensions (20 µL of the concentrated solution, see above) were deposited on a thin sheet of parafilm, and the polylysine/TetraSpeck coated coverslips were gently squeezed against the parafilm for 5 min at room temperature and rinsed twice. A depression slide was filled with 200 µL of pre-centrifuged Eternity buffer. The coverslip was gently placed face down above the cup-like depression to avoid bubbles. Excess buffer was delicately discarded and the coverslip was then sealed with twinsil. dSTORM acquisition was started within a couple of hours.

### Mounting centrosome slides for dSTORM imaging

After immunofluorescence labelling, samples were rinsed once with PBS and once with water. Similarly to HCVpp samples, samples were incubated with TetraSpeck beads before mounting with Eternity buffer containing MEA or BME (see section “Tetraspeck beads incubation”). Microscopic observation and image acquisition were started within a couple of hours (Day 0). Coverslips were then stored in the dark at 4 °C until further observation.

### 3D localisation with TetraSpeck using PRILM

Binding of the beads is necessary to avoid Brownian fluctuation. A 100 nm bead can fluctuate due to the looseness of the link between the bead and the surface and even small fluctuation (at the nm scale) can worsened the quality of the PSF recontruction. We used polylysine coating to bind the beads to the surface. However this process leads to different strengths in the bead attachment from loose to tight attached beads. In the optimal experiments, the loose attached beads were removed by the hydrodynamic wash of the petri dish with distilled water. We took advantage of the existence of the loose binding to generate a non-optimal PSF where the loose attached beads were not removed. The PSF generated, that mixed the PSF of the tight and loose attached beads was thus non optimal.

#### Preparation of microdishes with TetraSpeck

Microdishes with a high glass bottom (Ibidi dishes ref 81158, with a 170 µm ± 5 µm glass coverslip) were coated with polylysine (see HCVpp section “Polylysine coating”). Two different dishes processed as follows were used to generate a 3D localisation file.

#### Reference point spread function (PSF): tight binding

Ten microlitres of a suspension of TetraSpeck beads were added to the dried dish and dispersed using an 18 mm coverslip. After a 5 min incubation, the coverslip was removed and 3 washes with dH_2_O were performed. Mounting was performed with 400 µL Eternity buffer and a 30 mm round coverslip gently placed to avoid bubbles and sealed with twinsil.

#### Non-optimised PSF: loose binding

Ten microlitres of a suspension of TetraSpeck beads were added to the dried dish. Mounting was performed immediately with 400 µL of Eternity buffer and a 30 mm round coverslip, sealed with twinsil.

#### Acquisition of experimental PSF with 3D slider

A field of view containing at least 20 well dissociated fluorescent beads was selected (Supplementary Fig. 8a). After positioning the 3D slider in the optical path designed for PRILM^26^, the beads no longer appeared as single dots, but as double dots (Supplementary Fig. 8b). The focal plane was then adjusted to obtain a 45° angle between the horizontal line and the line going through both doublets. This step should be performed on a temperature-stabilised microscope (switched on 2 h in advance and equipped with a closed microscope chamber) to avoid drift during acquisition. A z-stack with piezo stage was acquired using the centre mode set on the 45° angle, with a 10 nm z-step and 401 sections with 45% of the 642 nm laser.

#### Generation of 3D localisation file

This operation was conducted according to manufacturer’s instructions for both conditions (tight binding and loose binding). The resulting mean PSFs were visualised in 3D (Supplementary Fig. 8b) and used to calculate two localisation precision files (Supplementary Fig. 8c).

### dSTORM acquisition

dSTORM acquisition was performed on an ELYRA PS1 microscope using the Zen software (Zeiss). Samples were observed with the 63x oil-immersion Plan APOCHROMAT objective lens (Zeiss, NA = 1.4) using a 1.6x lens. A full-size image in brightfield and fluorescence was acquired with an EMCCD camera (Andor iXon DU897, pixel size: 16 µm, 512 × 512). The focal plane was defined in fluorescence mode so that the donut-like shape of the LipoParticle was visible (insets to Supplementary Fig. 2), in order to start acquisition as close as possible to the equatorial plane of the LipoParticle. A pumping step was achieved for less than 30 s, in HILO illumination mode (52°) with ultra-high power using 70% of the 642 nm laser (150 mW nominal power), with the corresponding LP 655 + LP750 filter position. Acquisition of 20,000 images, using a ROI centered around the structure was achieved with a 20% laser power with the following camera parameters: 280 for EM-gain (max value at 300) and 30 Hz rate. Z stabilisation was achieved with the Definite Focus system (Zeiss) activated once every 300 images. Three series were performed for each time point (D_0_ and D_6_ for Fig. 1b, and D_0_, D_7_, D_14_, D_36_ and D_58_ for Fig. 1c) on the same dish, mounted either with classical or Eternity buffers and stored in the dark at 4 °C between acquisitions (Table 1).

**Table 1:** Conditions used to acquire and visualise images in Fig. 1.

Figure panel	Sample type	Imaging mode	Laser angle	Buffer* (age in day)	Support	# acquired images	Visualisation
1b	Blinking LipoP	2D dSTORM	HILO	Glox (0)	Small Wilco	15,000	IGOR
1c	Blinking LipoP	2D dSTORM	HILO	Glox (6)	Small Wilco	15,000	IGOR
1 d	Blinking LipoP	2D dSTORM	HILO	Eternity MEA (0)	Small Wilco	15,000	IGOR
1 e	Blinking LipoP	2D dSTORM	HILO	Eternity MEA (6)	Small Wilco	15,000	IGOR
1j	Blinking LipoP	2D dSTORM	HILO	Eternity MEA (0)	Small Wilco	15,000	IGOR
1k	Blinking LipoP	2D dSTORM	HILO	Eternity MEA (7)	Small Wilco	15,000	IGOR
1 l	Blinking LipoP	2D dSTORM	HILO	Eternity MEA (14)	Small Wilco	15,000	IGOR
1 m	Blinking LipoP	2D dSTORM	HILO	Eternity MEA (36)	Small Wilco	15,000	IGOR
1n	Blinking LipoP	2D dSTORM	HILO	Eternity MEA (58)	Small Wilco	15,000	IGOR

*pH of the buffer was set to 8.

For HCVpp samples (Fig. 2b–c), dSTORM acquisition was carried out under similar conditions as LipoParticles, with 10,000 images recorded *per* series (Table 2).

**Table 2:** Conditions used to acquire and visualise images in Fig. 2.

Figure panel	Sample type	Imaging mode	Laser angle	Buffer*(age in day)	Support	# acquired images	Visualisation
2a	Viruses	Widefield	Epi	Eternity MEA (0)	Small Wilco	1	B/W
2c	Individual virus 2b	2D dSTORM	TIRF	Eternity MEA (0)	Small Wilco	10,000	IGOR
2d	Individual virus	TEM	—	—	Grid	1	B/W
2e	Centrosome	Widefield	Epi	Eternity MEA (0)	Coverslip	1	B/W
2 f	Centrosome	2D dSTORM	HILO	Eternity BME (0)	Coverslip	100,000	Gaussian + centroid
2 g	Centrosome 2 f	2D dSTORM	HILO	Eternity BME (0)	Coverslip	100,000	IGOR
2 h	Centrosome	TEM	—	—	Grid	1	B/W
2i	Centrosome	2D dSTORM	HILO	Eternity BME (1)	Large Wilco	50,000	IGOR
2j	Centrosome	2D dSTORM	HILO	Eternity BME (2)	Large Wilco	50,000	IGOR
2k	Centrosome	2D dSTORM	HILO	Eternity BME (9)	Large Wilco	50,000	IGOR
2 l	Centrosome	2D dSTORM	HILO	Eternity BME (17)	Large Wilco	50,000	IGOR
2 m,n,o	Centrosome	2D dSTORM	HILO	Eternity BME	Large Wilco	50,000	Statistics

*pH of the buffer was set to 8.

For centrosome samples, dSTORM acquisition was carried out as for LipoParticles, with 50,000 images *per* series using an ROI centered around the structure (Table 2, Supplementary Table 1). For the reconstruction shown in Fig. 2f–g, 100,000 images were recorded to assay for the optimal number of images necessary in 2D (Supplementary Fig. 9b).

For 3D dSTORM, acquisition was carried out with the 3D slider in place (Supplementary Fig. 8), and 100,000 images were recorded *per* series for both LipoParticles (Fig. 3a–h and Supplementary Fig. 9a) and centrosome in epifluorescence mode (Fig. 3i–p and Table 3).

**Table 3:** Conditions used to acquire and visualise images in Fig. 3.

Figure panel	Sample type	Imaging mode	Laser angle	Buffer*	Support	# acquired images	Visualisation
3a,b,e,f	Blinking LipoP	3D dSTORM	Epi	GLOX	Small Wilco	100,000	gaussian
3c,d,g,h	Blinking LipoP	3D dSTORM	Epi	GLOX	Small Wilco	100,000	3D surface reconstruction
3i,j,m,n	Centrosome	3D dSTORM	Epi	Eternity	Coverslip	100,000	gaussian
3k,l,o,p	Centrosome	3D dSTORM	Epi	Eternity	Coverslip	100,000	3D surface reconstruction

*pH of the buffer was set to 8 and observed at D_0_.

For dual coloured dSTORM (Supplementary Fig. 7), acquisition of 50,000 images was first performed for far red fluorophores (AF647 or DL650), followed by 50,000 images for the red DL550 channel, using the 561 nm laser (200 mW nominal power), with the corresponding individual filter BP 570–650 + LP750 (Table 3, Supplementary Table 1).

### 2D dSTORM data analysis and visualisation

Data analysis was carried out with the Zen software (Zeiss) as follows: (i) gaussian fitting: peak mask size = 7 pix and peak intensity to noise = 8.5 (ignore overlap mode). Image sampling was set to ½ the measured localisation precision (i.e. 5 or 10 nm) in a given size ROI surrounding the structure. PSF filter was set between 120 to 280 nm to restrain depth of focus; (ii) drift correction: using the “model-based” set up at a maximum of 30 segments, based on an auto-correlation method; (iii) connexion: grouping of events using 5 frames for the maximum “ON” time, 1,000 frames for off gap and 1 pixel for capture radius. For multi-colour dSTORM, image registration was performed on the Zeiss software, after reconstruction, using the Tetraspeck beads as references.

The list of parameters provided the number of events *per* series, as well as the number of photons *per* event, and the median value was calculated for localisation precision of the series and shown with standard deviations on graphs. Visualisation was obtained by representing each event either by a single tiny dot at its gravity centre (centroid representation mode) or by a gaussian distribution with a fixed width (Fig. 2b,f). Moreover, IGOR representation allows to visualise the localisation precision of each point using a colour-coded rainbow scale (Fig. 2c,g). Their size is also proportional to the value of their localisation precision. For clarity, this ratio is adjusted on each figure from 1/1 to 1/10 between the smallest and the biggest point.

### 3D dSTORM data analysis and visualisation

3D analysis was carried out following the same steps as those for 2D analysis, with an additional step after the first one, to integrate the third dimension by selecting the localisation file previously generated (Supplementary Fig. 8c and section “Generation of 3D localisation file”). 3D reconstructions (Fig. 3c,d,g,h,k,l,o,p), were represented using the default surface rendering mode without Look Up Table (LUT). Supplementary Movies 1 to 6 were created with the Zen software tool, with the option “series” and accelerated 16 times (Supplementary Movies 1 to 6).

### dSTORM reconstruction encoded with IGOR

To overcome the issue of a representation with homogeneous dots, a routine was written using IGOR pro software (Wavematrics Inc). This software, based on matrix calculation, allows scientific graphing, data analysis, image processing and programming. A specific routine was developed for 2D representations (Supplementary File 1), in which localisation precision was encoded according to a colour and size code, to appreciate not only the position of an event but also its localisation precision. The colour code was represented with the inverted rainbow LUT. The absolute marker size was adapted according to the observed structure, and size variations between dots were indicated in the colour-coded scale. The Gizmo mode was used to provide a dotted 3D representation (Supplementary File 2), not available in the Zen software (surface or transparent modes only), in which the size of each dot is proportional to its x, y and z localisation precision (Supplementary Fig. 9a, right column).

## Supplementary information

Supplementary Information

Movie 1

Movie 2

Movie 3

Movie 4

Movie 5

Movie 6
