# Supplementary material for: Innovative particle standards and long-lived imaging for 2D and 3D dSTORM
Source: Sci Rep. 2019 Nov 29;9:17967. doi: 10.1038/s41598-019-53528-0 (PMC6884466; doi:10.1038/s41598-019-53528-0)
Supplement: Supplementary file 1 — Supplementary Information [file 41598_2019_53528_MOESM1_ESM.docx]

**Supplementary information**

**Innovative particle standards and long-lived imaging for 2D and 3D dSTORM**

Angelina Provost^a,b,§,^ Corentin Rousset^a,c,§,^, Laura Bourdon^a,b,d^, Sarra Mezhoud^a,d^, Emma Reungoat^a,c^, Camille Fourneaux^a,c^, Timothée Bresson^a,b,d^, Marine Pauly^a,d^, Nicolas Béard^a,c^, Laura Possi-Tchouanlong^a,b,d^, Boyan Grigorov^a,c^, Philippe Bouvet^a,b,c^, Jean-Jacques Diaz^a,c^, Christophe Chamot^a,f^, Eve-Isabelle Pécheur^a,c^, Catherine Ladavière^a,d,*^, Marie-Thérèse Charreyre^a,b,d,*^, Arnaud Favier^a,b,d^, Christophe Place^a,b,e,*#^, Karine Monier ^a,b,c,*#^

^§^ Co-first author

^#^ Co-senior authors

* Co-corresponding authors


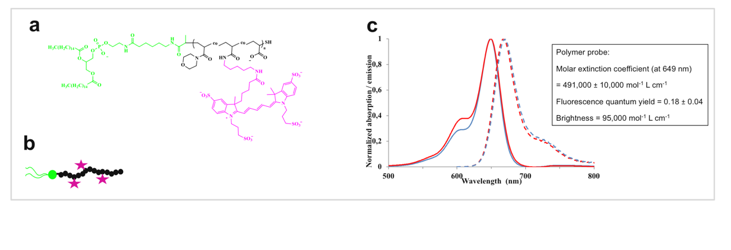
**Figure S1**: **Chemical structure and optical properties of the fluorescent lipid-polymer-AF647 probe**. (a) Chemical structure of the lipid-polymer-AF647 probe; (b) Schematic representation with the polymer chain (black, hydrodynamic radius in the 3 nm-range for a 20,000 g/mol polymer chain), the terminal lipid-group (green) and the covalently-bound AF647 fluorophores (purple stars, average of 3 fluorophores *per* polymer chain); (c) Normalized absorption (full line) and fluorescence emission (dashed line) spectra in water of the AF647 fluorophore (blue) and polymer probe (red), inset: photophysical characteristics of the polymer probe.

**Figure S2: Large field of view of LipoParticles.** LipoParticles labelled with the lipid-polymer-AF647 probe, observed in epifluorescence (a, red) and transmission (c, white) mode. Merge of both images shows the high labelling ratio of LipoParticles (b).


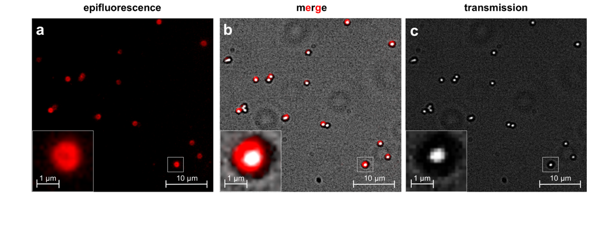


**Figure S3: Eternity buffer enables visualisation of LipoParticles at pH 5 and 8.** dSTORM reconstruction of LipoParticles labelled with lipid-polymer-AF647 probe in Eternity buffer at pH 5 (a) and 8 (b). Points are color-coded (5 to 60 nm; inverted rainbow scale) and size-coded (from a 1 to 2 ratio) as a function of the localisation precision. Number of blinking events, median localisation precision and average number of photons *per* events are indicated below each image. Supplementary Table 1 summarizes the conditions used to acquire and visualise images in this figure.


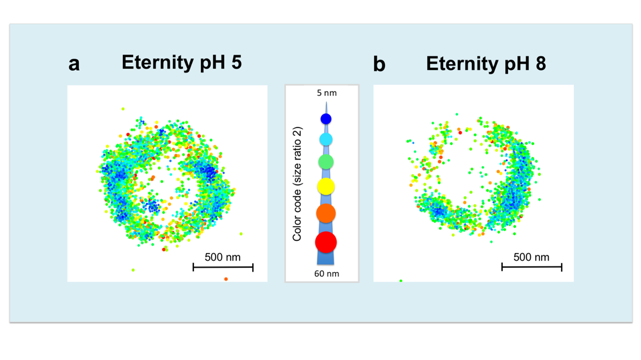


**Figure S4**: **Large field of view of HCVpp particles observed by TEM.** TEM images of HCVpp (negative staining) in the absence (a) or presence (b) of the lipid-polymer-AF647 probe, showing structural similarity.

**
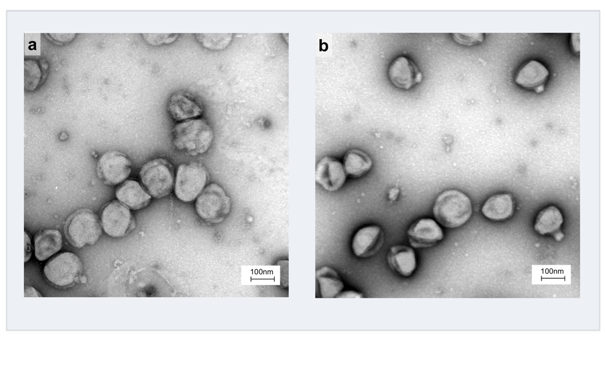
**

**Figure S5: Optimisation of Eternity robustness for dSTORM imaging.**

dSTORM reconstruction from density of points using the gaussian width (intensity varies from blue to white as a function of the density of points) of centrosomes from U2OS cells, labelled as indicated above and imaged in Eternity buffer with mercaptoethanolamine (MEA, a) or β-mercaptoethanol (BME, b). For these experiments, cells were plated on Willco dishes. A schematic drawing of the orientation of the centrosome is indicated as an insert in each image. [Bottom panel]: Number of blinking events (c), median number of photons *per* event (d) and median localisation precision (e) are presented in bar graph as a function of the reducing agent MEA (left, indigo blue, n=6, from 3 independent biological replicates) or BME (right, cyan blue, n=13, from 3 independent biological replicates). Standard deviation is indicated as an error bar for each condition. Student’s t test gives a p value of 0,19 (c), 0,016 (d) and 0,005 (e). (*) Significant differences between both populations with a 5% confidence interval. Supplementary Table 1 summarizes the conditions used to acquire and visualise images in this figure.


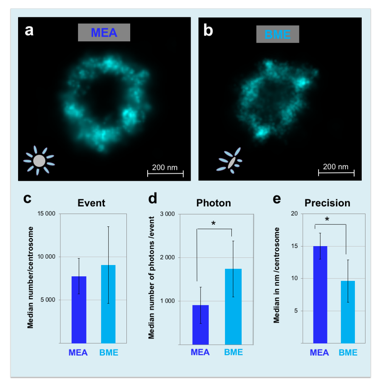


**Figure S6: Multicolour dSTORM imaging with the Eternity buffer.**

3D reconstructions acquired on 9 independent biological samples by 2-color dSTORM were obtained from 10,000 acquisition sequences performed on a centrosome. This was obtained after labelling Cep164 and pericentrin with secondary antibodies coupled to DL550, and AF647 or DL650, respectively. A typical 2-colour reconstruction obtained with these 2 labels is shown in Supplementary Fig. 7. Bar graph of the number of photons (median number/centrosome, a), the xy precision (median localisation/centrosome in nm, b) and the z precision (median localization/centrosome in nm, c) for DL650, DL550 and AF647 in classical buffer (orange, left) or Eternity buffer (blue, right). Standard deviations are represented as error bars for 2 centrosomes detected with DL650 on 2 different slides in classical buffer, and 6 centrosomes detected with DL650 on 2 different slides in Eternity buffer. For DL550, standard deviations represent error bars for 4 centrosomes detected on 3 different slides in classical buffer, and 8 centrosomes on 2 different slides in Eternity buffer. For AF647, standard deviations represent error bars for 2 centrosomes detected on 2 different slides in classical buffer, and 4 centrosomes on 3 different slides in Eternity buffer. Supplementary Table 1 summarizes the conditions used to acquire and visualise images in this figure.


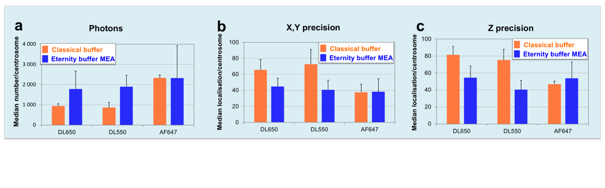


**Figure S7: Long-lived multicolour dSTORM imaging with Eternity buffer.** Centrosomes from RPE1 cells were detected with antibodies directed against Cep164 and pericentrin, using secondary antibodies respectively labelled with DL550 and AF647, and imaged in Eternity buffer after 40 days. Gaussian visualisation of 2 color dSTORM reconstruction of the whole field of view (a). Localisation precision maps were color-encoded with IGOR (b,c,d) using an inverted rainbow LUT to display pericentrin (b) and Cep164 (c). A merge representation of localization maps encoded with a cyan LUT for pericentrin and a red LUT for Cep164 was assembled and chromatic translation was applied using Tetraspeck beads as references (d). Reconstructions of three other centrosomes similarly labelled but oriented differently are shown (e). A centrosome schematic representation displaying the same orientation than in d was drawn to ease image comprehension (f). Supplementary Table 1 summarizes the conditions used to acquire and visualise images in this figure.


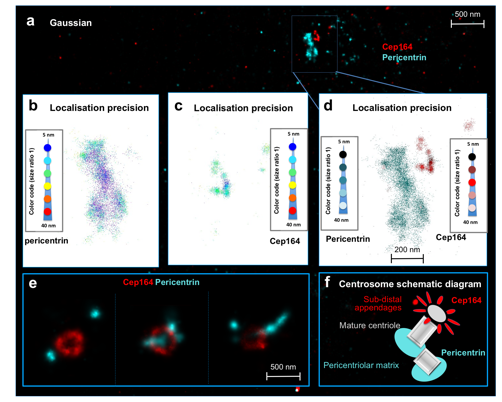


**Figure S8: Procedure for 3D dSTORM data generation and validation.** The procedure to collect 3D dSTORM data relies on 2 steps: 1) the first (purple panel) required 100 nm-fluorescent TetraSpeck beads tightly or loosely attached to a surface (a). The insertion of a 3D-slider in the optical path during the acquisition of z-stacks led to the acquisition of 2 different experimental PSF, using PRILM (b). These experimentally-acquired PSFs allowed the generation of 3D localisation files (c); 2) the second step (blue panel) is to mount LipoParticles in a Wilco dish with Eternity buffer and to seal with twinsil prior the observation of the deposited blinking LipoParticles (d) and the acquisition of a 3D dSTORM series on a single Lipoparticle (e). Finally 3D reconstruction is calculated with the 3D localisation files generated during the first step (with tight or loose binding, f).


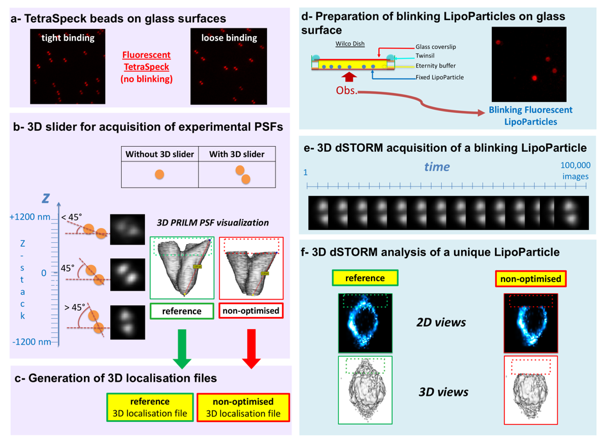


**Figure S9: Influence of the number of images on 3D and 2D reconstructions with different visualisation modes.** 3D (a) and 2D (b) dSTORM reconstructions of (a) a LipoParticle, and (b) distal appendages of a centrosome labelled as indicated above and imaged in Eternity buffer (same dataset as in Fig. 2f-g). Increasing numbers of image subsets from the same series were processed, using either the ZEN software to obtain reconstructions with a 3D surface rendering mode (a, left) and with a gaussian mode (b, left), or using the IGOR software to obtain 3D reconstructions, each purple point being scaled according to its localisation precision (a, right) or in a color-coded way indicated on the right (b, right). Supplementary Table 1 summarizes the conditions used to acquire and visualise images in this figure.


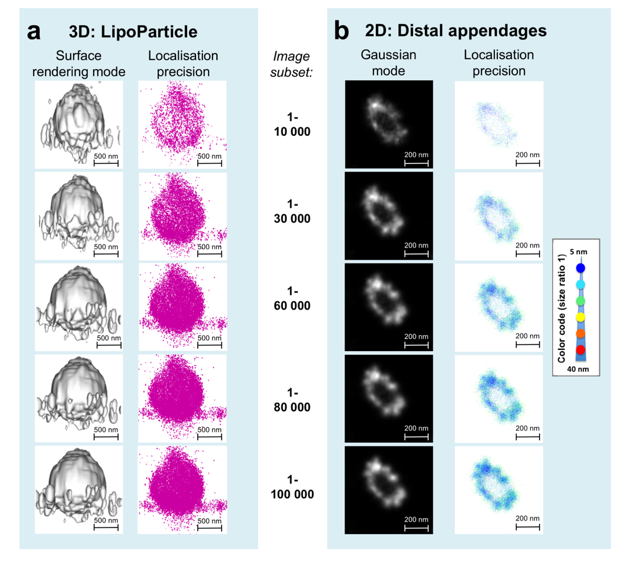


**Figure S10: Origin of centrosome replicates analysed on Fig. 2m-o.** Centrosome structures were collected from 3 independent coverslips. D is the time in days when image collection was performed after mounting the coverslips (cvs) with Eternity buffer (D_0_). Each square corresponds to one centrosome structure. Coverslips were kept sealed and at 4°C in the dark when not imaged. Reconstructed images of this kinetics can be found in Fig. 2i-l and statistics obtained with these structures are presented in Fig. 2m-o.


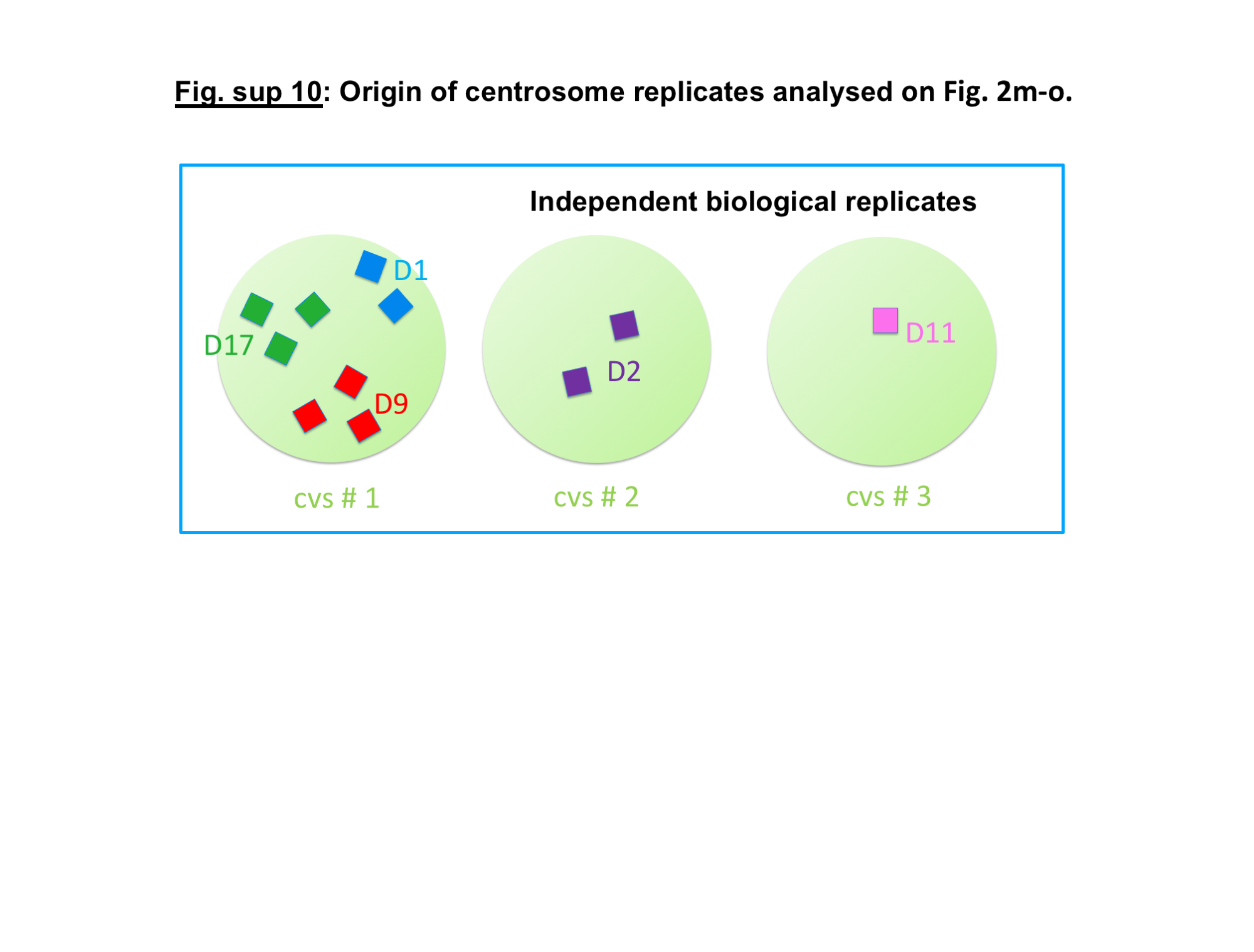


**Movie 1**: Animated 360° view of Fig. 3c, representing the 3D dSTORM reconstruction of a LipoParticle calculated with the non-optimised PSF, using a surface rendering mode performed with the ZEN software. The top part of the LipoParticle appears truncated in this reconstruction.

**Movie 2**: Animated 360° view of Fig. 3g, representing the 3D dSTORM reconstruction of a LipoParticle calculated with the reference PSF, using a surface rendering mode performed with the ZEN software. The top part of the LipoParticle clearly appears in this reconstruction.

**Movie 3**: Animated 360° view of Fig. 3k, representing the 3D dSTORM reconstruction of the distal appendages of a centrosome detected in U2OS cells calculated with the non-optimised PSF, using a surface rendering mode performed with the ZEN software.

**Movie 4**: Animated 360° view of Fig. 3l, representing the 3D dSTORM reconstruction of the distal appendages of a centrosome detected in U2OS cells calculated with the non-optimised PSF, using a surface rendering mode performed with the ZEN software and visualised sideways.

**Movie 5**: Animated 360° view of Fig. 3o, representing the 3D dSTORM reconstruction of the distal appendages of a centrosome detected in U2OS cells calculated with the reference PSF, using a surface rendering mode performed with the ZEN software.

**Movie 6**: Animated 360° view of Fig. 3p, representing the 3D dSTORM reconstruction of the distal appendages of a centrosome detected in U2OS cells calculated with the reference PSF, using a surface rendering mode performed with the ZEN software and visualised sideways.

**Supplementary Table 1:** Conditions used to acquire and visualise images in Supplementary Figures.

| **Figure panel** | **Sample type** | **Imaging mode** | **Laser angle** | **Buffer (pH)** | **Support** | **# acquired images** | **Visualisation** |
| --- | --- | --- | --- | --- | --- | --- | --- |
| Sup 3a | Blinking LipoP | 2D dSTORM | HILO | Eternity MEA (5) | Small Wilco | 15000 | Igor |
| Sup 3b | Blinking LipoP | 2D dSTORM | HILO | Eternity MEA (8) | Small Wilco | 15000 | Igor |
| Sup 5a | Centrosome | 2D dSTORM | HILO | Eternity MEA (8) | Large Wilco | 50,000 | Gaussian with cyan LUT |
| Sup 5b | Same centrosome as in 2i | 2D dSTORM | HILO | Eternity BME (8) | Large Wilco | 50,000 | Gaussian with cyan LUT |
| Sup 6a-c | Centrosome | 3D dSTORM 2 colors | HILO | GLOX or Eternity MEA (8) | Coverslip | 10,000 | Statistics |
| Sup 7a | Centrosome | 2D dSTORM | HILO | Eternity MEA (8) | Wilco | 50,000 each color | Gaussian |
| Sup 7b,c,d | Centrosome | 2D dSTORM | HILO | Eternity MEA (8) | Wilco | 50,000 each color | Igor |
| Sup 7e | Centrosome | 2D dSTORM | HILO | Eternity MEA (8) | Wilco | 50,000 each color | Gaussian |
| Sup 9a | Same blinking LipoP as in 3a-h | 3D dSTORM | Epi | GLOX (8) | Small Willco | 10,000 to 100,000 | 3D surface or Igor |
| Sup 9b | Same centrosome as in 2f | 2D dSTORM | HILO | Eternity BME (8) | Coverslip | 10,000 to 100,000 | Gaussian or Igor |

**Supplementary Files**

**Supplementary File 1: Specific routine developed for 2D representations in IGOR software and available as IGOR specific file on demand.**

#pragma rtGlobals=3 // Use modern global access method and strict wave access.

#include <MatrixToXYZ>

//index 0

//first frame 1

//number frame 2

// frame missing 3

//x 4

//y 5

//precision 6

//nbre photons 7

//background 8

//chi square 9

//psf 10

//

//channel 11

//zslice 12

//

//

macro PresentationSTORM()

variable ind, scolex, scoley, indmax, indmaxauto

string chemin, name1, lista, nom1, nom2, nom3, axil

killwaves/A/Z

make/D/O/N=(18, 9) resumeUpdate

//loading xls files

make/T/O TextWave={"LP3H003StormGlox-4H-b2-PALM.xls","LP3H003StormGlox-4H-d2-PALM.xls","LP3H003StormGlox-4H-f2-PALM.xls","LP3H003StormGlox-6D-b2-PALM.xls","LP3H003StormGlox-6D-d2-PALM.xls","LP3H003StormGlox-6D-f2-PALM.xls","LP3H003StormN2-4H-b2-PALM.xls","LP3H003StormN2-4H-d2-PALM.xls","LP3H003StormN2-4H-f2-PALM.xls","LP3H003StormN2-6D-b2-PALM.xls","LP3H003StormN2-6D-d2-PALM.xls","LP3H003StormN2-6D-f2-PALM.xls"}

newpath/O/Q path1, "C:Users:cplace:Desktop:LongLiveLipo:pH8"

newpath/O/Q path1ext, "C:Users:cplace:Desktop:LongLiveLipo:pH8:graphpreci"

indmax=DimSize(TextWave, 0)-1

//coding the STORM size and precision

ind=-1

do

ind+=1

XLLoadWave/R=(A1,M50000)/Q/D/N=wave/P=path1 TextWave[ind]

axil=num2str(ind+1)

nom1="ypos"+axil

nom2="xpos"+axil

nom3="preci"+axil

duplicate wave5 $nom1

duplicate wave4 $nom2

//choose precisions 6 ou photons 7

//duplicate wave6 $nom3

duplicate wave7 $nom3

display/K=1/N=graphepreci $nom1 vs $nom2

ModifyGraph mode=3,marker=19

//choose photons blue or precision size and rainbow

//ModifyGraph zmrkSize($nom1)={$nom3,*,*,1,10}

//red the smallest

//ModifyGraph zColor($nom1)={$nom3,0,180,Rainbow,0}

//black the biggest

ModifyGraph zColor($nom1)={$nom3,0,1000,blue,1}

ModifyGraph height={Aspect,1}

wavestats/Q $nom2

scolex=V_avg

wavestats/Q $nom1

scoley=V_avg

SetAxis left scoley-1000,scoley+1000;

SetAxis bottom scolex-1000,scolex+1000

Label bottom "précisions "+axil + TextWave[ind]

newpath/O/Q path1ext,"C:Users:cplace:Desktop:LongLiveLipo:pH8:graphphot"

//save picture

SavePICT/P=path1ext/O/E=-6/B=72 as "graphephot"+axil+".jpg"

while (ind<indmax)

end

**Supplementary File 2: Specific routine developed for 3D representations in IGOR software and available as IGOR specific file on demand.**

#pragma rtGlobals=3 // Use modern global access method and strict wave access.

#include <All Gizmo Procedures>

macro gizzy()

variable ind, indmax, taipt, preciH, preH, divi, divilog

string camino, camina, nono, nona, nomi, taiptstr, divistr, divilogstr, preciHstr

killwaves/A

make/T/O Nomdufish={"AP-057_647(2)_PALM_Crop-Im-buffer-Cellule-Filter-precision.txt", "AP-057_647(2)_PALM_Crop-Im-buffer-Filter-precision.txt", "AP-057_647(2)_PALM_Crop-Zeiss-Filter-precision.txt" }

camino= "C:Users:christophe:Desktop:centro3D"

camina= "C:Users:christophe:Desktop:centro3D:figureGizmo"

newpath/O/Q/C path1, camino

newpath/O/Q/C path2, camina

indmax=0

ind=-1

do

ind+=1

LoadWave/O/Q/A/W/G/H/P=path1 Nomdufish[ind]

duplicate/O Position_X, volume

volume=2* (Precision_XY)^3 + (Precision_Z)^3

//General text load from "AP-057_647(2)_PALM-PSFCell-cropA.txt"

//Data length: 10360, waves: Position_X, Position_Y, Position_Z, Precision_XY, Precision_Z

divi=500

Concatenate/O{Position_X,Position_Y ,Position_Z},pos3Dcep

Concatenate/O{Precision_XY,Precision_XY,Precision_Z},pre3Dcep

Duplicate/O pre3Dcep,pre3Dcep500

//Duplicate pre3Dcep,pre3Dcep100

//Pre3Dcep1000=pre3Dcep/1000

Pre3Dcep500=pre3Dcep/divi

Duplicate/O pre3Dcep,pre3Dceplog

pre3Dceplog=log(pre3Dcep)

Duplicate/O pre3Dceplog,pre3Dceplogdiv

divilog=30

pre3Dceplogdiv=pre3Dceplog/divilog

preciH=100

duplicate/O pre3Dcep pre3DcepLim

pre3DcepLim= (pre3Dcep[p][q][r]>preciH)? preciH : pre3Dcep[p][q][r]

duplicate/O pre3DcepLim pre3DcepLimdiv

pre3DcepLimdiv=pre3DcepLim/divi

duplicate/O Precision_XY Preci_XYlim

Preci_XYlim = (Precision_XY[p]>preciH) ? preciH : Precision_XY[p]

Make/O/N=(512,512) L2Dmatrix, countmatrix

imagefromXYZ/AS {Position_X, Position_Y, Position_Z}, L2Dmatrix, countmatrix

duplicate/O L2Dmatrix, L2Dmatrixnew

//size limitation

pre3DcepLim= (pre3Dcep[p][q][r]>preciH)? preciH : pre3Dcep[p][q][r]

wavestats/Q Position_X

wavestats/Q Position_Y

wavestats/Q Position_Z

nomi="giz"

//making

NewGizmo/I/K=1/N=Gizmo0/T=nomi/W = (750,0,1350,600)

AppendToGizmo/D scatter=pos3Dcep , name=scatter0

AppendToGizmo/D Axes=BoxAxes, name=axe0

ModifyGizmo startRecMacro

//functions

ModifyGizmo infoWindow = {600,450,1200,630 }

ModifyGizmo aspectRatio = 1

ModifyGizmo makeColorWave = {Preci_XYlim, Rainbow, 1}

ModifyGizmo makeColorWave = {pre3Dceplog, Rainbow, 1}

ModifyGizmo autoScaling = 1

ModifyGizmo setouterbox={0,1000,0,1000, -1000, 1000}

//operations

ModifyGizmo opName=ortho0, operation=ortho, data={-0.8, 0.8,-0.8,0.8,2,- 2}

taipt=0.05

ModifyGizmo modifyObject=scatter0, objectType=scatter,property={scatterColorType, 1}

ModifyGizmo ModifyObject=scatter0 property={ markerType,0}

ModifyGizmo ModifyObject=scatter0 property={ sizeType,1}

ModifyGizmo modifyObject=scatter0,objectType=scatter, property={sizeWave,pre3Dceplogdiv}

ModifyGizmo modifyObject=scatter0,objectType=scatter, property={colorWave,Preci_XYlim_C}

Sleep/s 1

ZeroToNaN(L2Dmatrix)

ModifyGizmo endRecMacro

taiptstr=num2str(taipt)

divistr=num2str(divi)

divilogstr=num2str(divilog)

preciHstr=num2str(preciH)

nona=removeending(Nomdufish[ind],".txt")

print nona

nono=nona+ "_" +"precisizelog_"+divilogstr+"_Rain1limcolor_"+preciHstr+ ".bmp"

ExportGizmo /P=path2 as nono

while (ind<indmax)

end

function ZeroToNaN(inwave)

wave inwave

inwave = (inwave[p][q]==0) ? NaN : inwave[p][q]

end
